# Multisensory, Nature-Inspired Recharge Rooms Yield Short-Term Reductions in Perceived Stress Among Frontline Healthcare Workers

**DOI:** 10.3389/fpsyg.2020.560833

**Published:** 2020-11-19

**Authors:** David Putrino, Jonathan Ripp, Joseph E. Herrera, Mar Cortes, Christopher Kellner, Dahlia Rizk, Kristen Dams-O’Connor

**Affiliations:** ^1^ Department of Rehabilitation and Human Performance, Icahn School of Medicine at Mount Sinai, New York, NY, United States; ^2^ Office of Well-Being and Resilience, Icahn School of Medicine at Mount Sinai, New York, NY, United States; ^3^ Department of Neurosurgery, Icahn School of Medicine at Mount Sinai, New York, NY, United States; ^4^ Division of Hospital Medicine, Mount Sinai Beth Israel, New York, NY, United States; ^5^ Department of Neurology, Icahn School of Medicine at Mount Sinai, New York, NY, United States

**Keywords:** COVID-19, stress, burnout, trauma, relaxation, biophilic design

## Abstract

We are currently facing global healthcare crisis that has placed unprecedented stress on healthcare workers as a result of the coronavirus disease 2019 (COVID-19). It is imperative that we develop novel tools to assist healthcare workers in dealing with the significant additional stress and trauma that has arisen as a result of the pandemic. Based in research on the effects of immersive environments on mood, a neuroscience research laboratory was rapidly repurposed using commercially available technologies and materials to create a nature-inspired relaxation space. Frontline healthcare workers were invited to book 15-min experiences in the Recharge Room before, during or after their shifts, where they were exposed to the immersive, multisensory experience 496 Recharge Room users (out of a total of 562) completed a short survey about their experience during an unselected, consecutive 14-day period. Average self-reported stress levels prior to entering the Recharge Room were 4.58/6 (±1.1). After a single 15-min experience in the Recharge Room, the average user-reported stress level was significantly reduced 1.85/6 (±1.2; *p* < 0.001; paired *t*-test). Net Promoter Score for the experience was 99.3%. Recharge Rooms such as those described here produce significant short-term reductions in perceived stress, and users find them highly enjoyable. These rooms may be of general utility in high-stress healthcare environments.

## Introduction

The coronavirus disease 2019 (COVID-19) pandemic in New York City led to surges of critically ill patients into hospitals that were already operating at or above capacity. Exceptional in the lifetimes of most hospital workers, this rapid influx required physicians, nurses, and other clinicians to endure extreme workloads in unfamiliar practice environments. There were shortages in personal protective equipment and other supplies, and many practitioners and support staff were redeployed from usual duties to serve on the frontlines caring for COVID-19 patients. Hospital workers were facing tremendous stress, all while navigating severe disruptions to daily life outside of work. Sources of stress, anxiety, and fear ranged from tangible to abstract: closure of schools, loss of childcare, economic hardship, fear of contracting the virus, fear of spreading the virus to loved ones, loss of patients, family members and coworkers to COVID-19, concern regarding one’s ability to perform new duties with minimal training, existential concerns about moral duties and responsibilities, and uncertainty regarding the future (Albott et al., 2020; Blake et al., 2020).

The confluence of these factors can impose moral suffering, fear, outrage, disgust, and depletion among health care workers (Patel et al., 2018) who may feel unprepared, unprotected, and unheard (Shanafelt et al., 2020). Moral injury, defined as the experience of “perpetrating, failing to prevent, bearing witness to, or learning about acts that transgress deeply held moral beliefs and expectations” (Litz et al., 2009; Currier et al., 2015), is often discussed in the context of war and combat, but these ideas are now being invoked in the language used by healthcare workers describing their responses to the current pandemic (Bai et al., 2004; Albott et al., 2020; Shanafelt et al., 2020). A recent survey found that healthcare workers at a large medical center in Baltimore, Maryland reported moral injury severity similar to that of military service members who served 7-month deployments in war zones, with particularly notable similarities in feelings of betrayal by others (Hines et al., 2020).

The World Health Organization (WHO) has recognized that protecting the mental health and well-being of healthcare workers, particularly those serving on the front lines, is essential for ensuring the immediate and long-term capacity of the healthcare community (McAlonan et al., 2007; World Health Organization, 2020). Absent a public health crisis such as the COVID-19 global pandemic, approximately 50% of physicians are experiencing burnout. Burnout was first described by Freudenberger (1971) as emotional depletion combined with exhaustion, real or perceived inefficacy, emotional lability, and psychosomatic symptoms that most often occurs in care settings requiring long hours, personal involvement, and empathy (Reith, 2018). Employee burnout has an extensive and well-documented negative impact on health care systems and organizations (Patel et al., 2018; Shanafelt et al., 2020). Absent a public health crisis such as the COVID-19 global pandemic, approximately 50% of physicians are experiencing burnout. Given the potential consequences on the emotional well-being of the workforce and overall care quality (Panagioti et al., 2018), the current need for brief, feasible, and scalable interventions to promote health care worker wellness and resilience is unparalleled. Ideally these interventions would promote readiness, another term borrowed from the military, which reflects the reality that frontline workers are needed to return to duty and ready to work at high levels of cognitive and physical performance (Nindl et al., 2018).

Healing environments designed to reduce stress and increase control in patients can result in less need for pain medication, fewer medical errors, better sleep, and improved outcomes (Parsons and Hartig, 2000; Zimring et al., 2004). A growing body of research indicates that virtual reality applications, particularly those that involve immersive architectural environments with visual and auditory manipulations, can directly impact emotions and their concordant psychophysiological responses (Badia et al., 2019). Consistent with the notion that humans are innately connected to nature, exposure to virtual environments that incorporate biophilic stimuli can lower physiological stress indicators, such as blood pressure and heart rate (Yin et al., 2019). Some evidence suggests that augmented reality manipulations to the built environment in urban environments may augment stress levels in urban environments in particular (Kalantari, 2016). To our knowledge, healing environments have not been widely implemented or investigated in frontline healthcare workers treating patients with severe acute respiratory syndrome coronavirus 2 (SARS-CoV-2).

In the field of cognitive neuroscience, the ability to maintain focus on a task or set of environmental stimuli is often referred to as “directed attention” and is thought of a finite cognitive resource that can be depleted (Vohs et al., 2014; Ohly et al., 2016). Directed attention fatigue (DAF) results in cognitive difficulties, poor decision making, emotion dysregulation, and performance variability during attentional tasks (Linden et al., 2005; Ohly et al., 2016). Attention restoration theory (ART) is a concept that has gained momentum in the field of environmental psychology, which postulates that DAF can be overcome by exposure to scenes depicting rich natural environments or direct exposure to nature (Kaplan, 1995). According to ART, a major goal of creating a restorative environment is to create scenes that encourage “soft fascination,” a cognitive state where one’s attention is held by the scene that they are taking in, but in a way that still permits reflection and the ability to address lingering, unresolved thoughts (Basu et al., 2019). During the initial 2020 surge of SARS-CoV-2 cases in the United States, our team developed and created multisensory, nature-inspired Recharge Rooms in a New York City hospital and made them available to essential staff. Design of these rooms followed the principles of ART to create experiences of soft fascination for users with the intention of creating moments of stress relief and relaxation. Here, we report initial user responses to the Recharge Room experience.

## Materials and Methods

**Figure 1:**
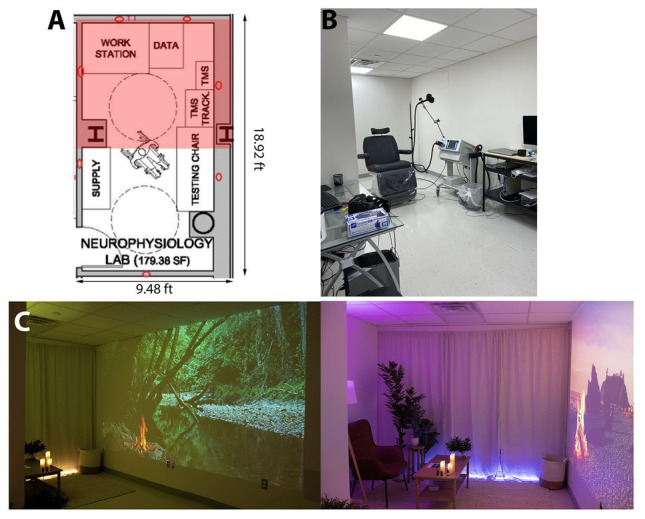
**(A)** Architectural plans of a candidate room to be repurposed as a recharge room. The pink shading represents the part of the room that is visible in the photographs of the space. **(B)** The room prior to transformation in its usual use-case as a neurophysiology testing space. **(C)** The finished Recharge Room showing two different scenes that are available to healthcare workers.

We rapidly converted under-utilized research laboratory space in a New York City hospital into custom-designed “Recharge Rooms” to provide an opportunity for health care workers to rest and refresh themselves during or after their shift. The Recharge Rooms were designed by following the principles of ART (Korpela and Hartig, 1996; Sahlin et al., 2016), with a specific focus on creating natural scenes and experiences that shift users away from states of directed attention and promoted states of soft fascination (Kaplan, 1995; Kaplan and Berman, 2010). Since soft fascination is often most easily elicited by scenes of nature (Basu et al., 2019), the resultant rooms created multisensory (visual, auditory, and olfactory), nature-inspired experiences, as these have also previously been found to confer physiological benefits (Maxwell and Lovell, 2017). These environments include silk imitation plants, projected scenes of soothing natural landscapes, low lighting that is tailored in color to match the projected landscapes, high definition audio recordings of nature sounds paired with relaxing music, and an infusion of essential oils and calming scents using an essential oil diffuser. The first candidate room selected for transformation into a Recharge Room was a rectangular, 179.38 square foot neurophysiology laboratory (Figure 1A). Four adults with moderate technical knowledge of the operation of consumer electronic devices such as HD projectors, WiFi technology, Google Home, Bluetooth speakers, and Hue Bridge automatic lighting systems spent approximately 4 h transforming this existing hospital space (Figure 1B) to a functional Recharge Room (Figure 1C).

All materials that were used for the transformation were easily sourced from online vendors. The user experience was designed to be voice-activated using Google Home, allowing visiting healthcare workers to activate the projector to screen different natural scenes on a blank wall in the room without having to interact with screens or touch any items in the room, minimizing user interaction with any surfaces. The Hue Bridge lighting system was programmed to synchronize with the different nature scenes being projected in the room (i.e., Hue lights would turn blue for ocean scenes and green for forest scenes). All materials are non-porous and can be quickly sanitized after each use for infection control purposes. Yuzu, hinoki, roman chamomile, and lavender essential oils were used to create scent profiles that were associated with the visualization of different natural scenes using an essential oil diffuser in one corner of the room. These essential oils were selected based on existing literature showing their efficacy in producing stress relieving and soothing effects (Matsumoto et al., 2014; Ali et al., 2015; Ikei et al., 2015). The silk imitation plants that were used to create the impression of a green space in the hospital room were arranged in a semi-circular pattern in behind the arranged seating that was available in the room. This was done to create the impression being fully immersed and surrounded by a natural environment.

Information about the Recharge Rooms, located at Mount Sinai Hospital, with a description of the overall environment and the hours of operation (4:30am–10pm daily), was distributed to staff *via* a website curated by the Icahn School of Medicine at Mount Sinai’s Office of Resilience and Well-being in partnership with the Mount Sinai Health System’s COVID-19 Staff Response. Frontline healthcare staffs were invited to book 15-min recharge experiences online to prevent crowding and breaching of social distancing protocols.

Prior to entering the recharge space for their scheduled appointment, users were prompted to complete a single-item Likert-style measure of perceived stress that was purpose-developed by the study team (Question 1, Table 1). Upon completion of a 15-min experience in the Recharge Room, users were again prompted to complete a measure of their perceived stress levels (Question 2, Table 1), and the Net Promoter Score (NPS), a well-validated measure of user experience (Question 3, Table 1; Reichheld, 2003). Finally, respondents were given the option of providing additional comments in an open-ended “additional comments” section prior to submission of the online survey form (Question 4, Table 1). Survey data gathered from all users during a consecutive 4-day period of general operation are presented here. We calculated descriptive statistics, conducted a paired *t*-test to quantify changes in stress levels, and calculated a NPS. All analyses were conducted in MATLAB version R2019b (Mathworks, Natick, MA).

**Table 1:** User experience questionnaire characteristics.

Question (response range)	Lower anchor	Upper anchor
*What was your stress level like when you walked in?* (1–6)	Not stressed at all	Extremely stressed
*What is your stress level like after your experience?* (1–6)	Not stressed at all	Extremely stressed
*How likely are you to recommend this experience to a friend or colleague?* (0–10)	Not at all willing	Very willing
*Any additional comments?* (N/A)	N/A	N/A

## Results

**Figure 2:**
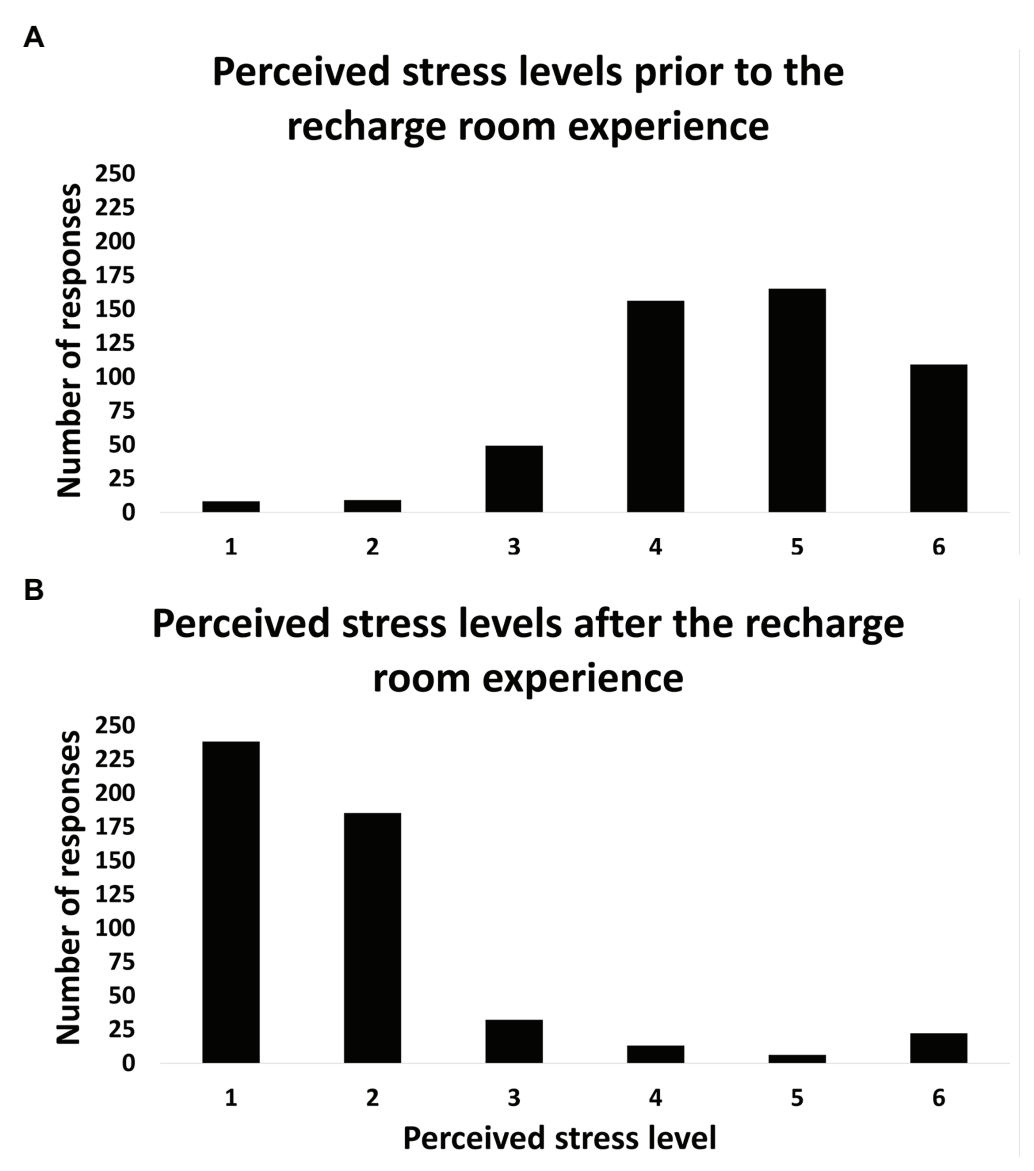
Bar graphs showing the distributions of perceived stress ratings of healthcare workers before **(A)** and after **(B)** a 15-minute experience in the Recharge Room.

Two hundred and nineteen frontline healthcare workers who requested use of the space during an unselected consecutive 14-day period completed the survey (out of a total of 562 healthcare workers who scheduled time to visit the space). At the time of data collection, the hospital had already admitted and managed 6,690 COVID-positive inpatients, with 1,034 of these requiring intubation and ventilator management. The surge continued throughout the data collection period, with hospital staff admitting more than 600 COVID-positive cases daily, and ventilator utilization was at nearly 70% of the hospital’s capacity. Prior to entry into the Recharge Room, average stress level was reported as 4.6/6 (±1.1). After a single 15-min experience in the Recharge Room, the average user-reported stress level was 1.85/6 (±1.2), representing an average 59.6% reduction in self-reported stress levels (Figure 2; *p* < 0.001; paired *t*-test).

The NPS for the experience was 99.3%, with 100% of respondents identifying as “promoters” (scores ranging between 8 and 10) of the experience.

A total of 207/496 respondents submitted qualitative feedback *via* the open-ended “additional comments” question. These qualitative comments were universally positive, such as “*This is wonderful!*” or “*This is such a needed and appreciated space at this time. It would be great if something similar could remain when this new normal is over*.” Additionally, several comments suggested that users viewed the experience as a gesture of institutional support, e.g., “*This is amazing! It’s a nice way for the system to show support for <hospital> employees!*”

## Discussion

Results from this program evaluation illustrate dramatic reductions in perceived stress, following brief exposure to a multisensory immersive Recharge Room. These findings support the utility of this low-cost, readily scalable support space for health care workers providing frontline care during the COVID pandemic. Open-ended written responses and spontaneous verbal feedback suggest that the Recharge Room influenced some of the key contributors to healthcare worker burnout (West et al., 2018) as well as the common primary endpoints of structured wellness intervention efforts (Panagioti et al., 2017).

The COVID-19 pandemic has placed stress on individual health care workers that is unprecedented for most, and the relationships between these feelings of moral suffering, exhaustion, fear, and stress are not known. The factors contributing to distress among COVID-19 healthcare workers may be somewhat unique, such as the anticipatory anxiety that may precede deployment to a COVID unit among clinicians assigned to COVID units, widespread supply shortages (Adams and Walls, 2020) that necessitate impossible choices between personal safety and patient care, and the expectations to perform tasks outside of one’s training or expertise which creates moral dilemmas unlike those encountered even in high stakes clinical care settings (McAndrew et al., 2018). Results of the current evaluation, therefore, may not generalize to the healthcare worker stress and anxiety experienced absent a global pandemic.

The current program evaluation project lacks the rigor of a structured clinical trial, and the use of a single-item self-report state stress measures as opposed to well-validated measures of burnout represent clear limitations of this work. Future research in a carefully controlled trial using a broader battery of validated self-report measures alongside physiological indices of stress response, as is standard in environmental psychology research, is warranted. Despite the impressive reductions in stress demonstrated here, the maintenance of these effects requires further investigation. In addition, use of the NPS as a standardized and well-validated measure of user experience was appropriate in the setting and scope of this program evaluation; however, there are limitations in how much such a short form can measure. Thus, while our NPS findings indicated that all Recharge Room users considered themselves to be “promoters” of the experience, in further research, we will conduct a more detailed user experience assessment in order to identify the specific aspects of the experience that create the strongest responses in the average user. This will allow us to identify ways in which to enhance the experience for future users.

There exists only limited evidence for the effectiveness of interventions designed to address stress and burnout in healthcare workers, though the need for such interventions is widely recognized (Marine et al., 2006). Recharge Rooms such as those described herein may facilitate short-term alleviation of distress experienced by frontline responders to the COVID pandemic.

## Data Availability Statement

The raw data supporting the conclusions of this article will be made available by the authors, without undue reservation.

## Ethics Statement

Ethical review and approval were not required for the study on human participants in accordance with the local legislation and institutional requirements. Written informed consent for participation was not required for this study in accordance with the national legislation and the institutional requirements.

## Author Contributions

DP, JH, MC, DR, and CK contributed to the conception and design of the Recharge Rooms. DP designed the questionnaire, collected the data, and performed the statistical analysis. DP, JR, and KD-O wrote the first draft of the manuscript. All authors contributed to the article and approved the submitted version.

### Conflict of Interest

The authors declare that the research was conducted in the absence of any commercial or financial relationships that could be construed as a potential conflict of interest.
